# Urgent Need to Understand and Prevent Gonococcal Infection: From the Laboratory to Real-World Context

**DOI:** 10.1093/infdis/jiae289

**Published:** 2024-05-31

**Authors:** Yara Ruiz García, Jeanne Marrazzo, Federico Martinón-Torres, Kimberly Workowski, Giulia Giordano, Mariagrazia Pizza, Woo-Yun Sohn

**Affiliations:** GSK, Tres Cantos, Spain; University of Alabama at Birmingham, Birmingham, Alabama, USA; Translational Pediatrics and Infectious Diseases, Hospital Clínico Universitario de Santiago de Compostela, Santiago de Compostela, Spain; Genetics, Vaccines and Infections Research Group, Instituto de Investigación Sanitaria de Santiago, University of Santiago de Compostela, Santiago de Compostela, Spain; Centro de Investigación Biomédica en Red de Enfermedades Respiratorias, Instituto de Salud Carlos III, Madrid, Spain; Emory University, Atlanta, Georgia; GSK, Siena, Italy; GSK, Siena, Italy; GSK, Rockville, Maryland, USA

**Keywords:** gonorrhea, *Neisseria gonorrhoeae*, immunology, *Neisseria meningitidis*, vaccine

## Abstract

*Neisseria gonorrhoeae* is widespread globally. Primary prevention is unsuccessful and antimicrobial resistance threatens optimal management. There is no specific vaccine and natural infection studies show that *N gonorrhoeae* can avoid and suppress immune responses. In addition to extensive variation in expression and specificity of many gonococcal surface antigens, it induces a robust inflammatory response through the Th17 pathway with a large influx of neutrophils and inflammatory cytokines but evades macrophages. The Th1- and Th2-mediated response is suppressed, resulting in low, short-lived antibody titers. Real-world evidence suggests that gonorrhea cases are reduced among recipients of *Neisseria meningitidis* group B vaccines containing outer membrane vesicles (OMVs). Although the first randomized trial of an OMV-containing MenB vaccine against *N gonorrhoeae* infection did not show statistically significant vaccine efficacy, ongoing trials might shed further light. Several candidate vaccine antigens for a gonococcal-specific vaccine are being evaluated preclinically but only one has reached clinical trials.

## Teaser

Gonorrhea is increasing in incidence globally; however, there is no vaccine available. This manuscript provides a description of the response of the immune system against gonococcal infections and potential vaccine mechanism of action to inform research and policy decisions.

*Neisseria gonorrhoeae* is widespread globally, with an estimated 82 million new infections in individuals 15–49 years of age during 2020 [1]. Most gonococcal infections occur in low- and middle-income countries, with the highest incidence in low-income countries [1]. In developed countries, as with other sexually transmitted infections (STIs), the highest incidence is seen in adolescents and young adults, ethnic minority groups, and those of low socioeconomic status [2, 3]. Other populations at particular risk of gonococcal infection include people with multiple sexual partners, people receiving preexposure prophylaxis (PrEP) therapy for human immunodeficiency virus, men who have sex with men, and people engaging in transactional sex [3, 4]. Gonococcal infection is increasing in incidence [3, 4]. Primary prevention measures (sexual abstinence or condom use) are not successful, and increases in antimicrobial resistance threaten the effectiveness of antibiotic treatment [3, 4].

Unfortunately, there is no specific vaccine against gonococcal infection and there have been numerous challenges associated with the development of an effective vaccine. The classical approach to vaccine design is to mimic natural infection; however, the ability of *N gonorrhoeae* to avoid and suppress the immune response means that natural infection does not reliably induce immunity [5]. In addition, there have been difficulties in implementing vaccine clinical trials due to frequent asymptomatic infections (notably in the pharynx and rectum), a limited infrastructure for providing care related to STIs, and social stigma related to STIs that can impact successful participation [5].

We have conducted a narrative review with the purpose of assessing available evidence on gonococcal infection and the impact of the closely related *Neisseria meningitidis* group B vaccine in preventing gonorrhea.

## WHAT WE KNOW ABOUT THE IMMUNE RESPONSE TO *N GONORRHOEAE*

**Figure 1.**
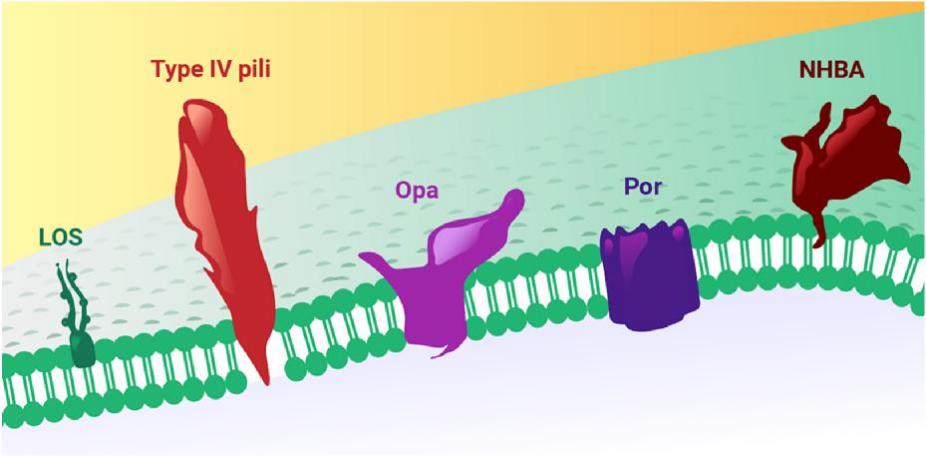
Important antigens on the surface of *Neisseria gonorrhoeae.* Abbreviations: LOS, lipooligosaccharide; NHBA, neisserial heparin binding antigen; Opa, opacity protein; Por, porin.

The first stage in infection with *N gonorrhoeae* is bacterial adhesion to the mucosal epithelium, mediated through surface antigens including lipooligosaccharide (LOS), type IV pili, opacity proteins, and the major outer membrane protein, porin (Por) (Figure 1) [5]. These surface antigens have an exceptional degree of variability as a result of genetic transformation [5]. In addition, *N gonorrhoeae* can reversibly switch the expression of proteins on and off in a process known as phase variation [5]. Together, these activities give the bacterium an unparalleled ability to evade the immune system.

Low titers of strain-specific antigonococcal local and systemic antibodies have been detected in men and women with uncomplicated genital infections [6]. History of previous infections does not increase antibody titers, indicating no induction of immunological memory [6]. Increased titers of anti-LOS antibodies have been seen in men experimentally infected with *N gonorrhoeae*, but there was no evidence of immunological memory upon reinfection [7]. In contrast, there is a strong inflammatory response associated with gonococcal infection, manifested by a large influx of neutrophils [8] and high levels of inflammatory cytokines such as interleukin (IL) 8, IL-6, IL-1β, and tumor necrosis factor [9].

Despite the large influx of neutrophils upon *N gonorrhoeae* infection, lack of immunoglobulin G (IgG) antibodies and inhibition of complement activation limit uptake of *N gonorrhoeae* by neutrophils [5, 10]. Intracellular killing is resisted by mechanisms including suppression of oxidative burst, resistance to nonoxidative killing mechanisms, and production of lytic transglycosylases [5, 11–13]. It is as yet unclear whether the neutrophil-driven inflammatory response to gonococcal infection benefits the bacterium or the host [14].

Complement inactivation and down-regulation are also believed to play a major role in the mechanisms by which *N gonorrhoeae* evades the immune response. The complement cascade is inactivated by binding of the gonococcal LOS to C3b via lipid A [15]. *Neisseria gonorrhoeae* uses its outer membrane Por molecules to bind the complement down-regulating proteins, C4b-binding protein and factor H, to evade killing by human complement [16]. In addition, sialylation of gonococcal LOS enables *N gonorrhoeae* to bind factor H [16]. Factor H usually acts to protect cells, as host structures bound to it are recognized as part of the host [15]. Indeed, sialylation of LOS inhibits all 3 complement pathways [17].

It is becoming increasingly recognized that the T-helper (Th) 17 proinflammatory response is promoted during gonococcal infection, suppressing cell-mediated Th1 and Th2 immune responses. Th17 cells characteristically produce IL-17 and it has been suggested that Th17 might have evolved to protect against pathogens that Th1 and Th2 responses are not effective against [18]. A study in patients with gonorrhea found that IL-17A, IL-23, and interferon-γ were significantly elevated compared with healthy individuals [19]. In another study, women with *N gonorrhoeae* or *Chlamydia trachomatis* infections had higher genital concentrations of cytokines, including IL-17, than women with no STI [20]. The same researchers also reported significantly higher levels of IL-17 specifically in the genital secretions of women with *N gonorrhoeae* or *C trachomatis* compared with women with no STI [21].

In mice, *N gonorrhoeae* has been shown to induce Th17 responses that promoted an influx of neutrophils and also recruited other innate defense mechanisms [22]. In contrast, it suppressed Th1- and Th2-dependent adaptive responses through induction of transforming growth factor beta (TGFβ) [23]. Importantly, treatment of mice with anti-TGFβ antibody during primary infection shifted the immune response away from Th17 toward Th1 and Th2 responses, promoted clearance of *N gonorrhoeae*, and aided development of Th1-dependent immune memory [24]. Based on these findings, it has been proposed that *N gonorrhoeae* promotes Th17 responses that it can largely survive but suppresses Th1 and Th2 responses that might be able to eradicate it, suggesting that vaccine development should be based on strategies eliciting Th1- or Th2-dependent immune responses [24].

Host risk factors and infection states also play a role in the immune response to *N gonorrhoeae*. There are major differences between men and women in the cellular lining of the urogenital tract, with different molecules acting as receptors for *N gonorrhoeae* and thus different mechanisms by which the gonococcus initiates infection in the urogenital tract [25]. Genetic factors influencing host susceptibility to gonococcal infection might also play a role. This has been observed for *N meningitidis* for which a number of mutations are associated with increased susceptibility to disease and more severe outcomes; such mutations include those resulting in increased bacterial adhesion and reduced complement function [26, 27]. However, to our knowledge, genetic factors influencing host susceptibility to *N gonorrhoeae* have not yet been identified.

It has been suggested that the adaptive immune response to *N gonorrhoeae* might depend on tissue penetration by the bacterium [5], based on the observations that women with severe pelvic inflammatory disease (PID) have higher titers of antibodies [28] and are protected against recurrence of PID [29]. This would explain why most gonococcal infections of the lower urogenital tract, pharynx, and rectum, which are largely asymptomatic or cause little sustained tissue damage, are not associated with an immunologic memory response [5].

## EVIDENCE OF PROTECTION AGAINST *N GONORRHOEAE* WITH VACCINES AGAINST MENINGITIS B

**Figure 2.**
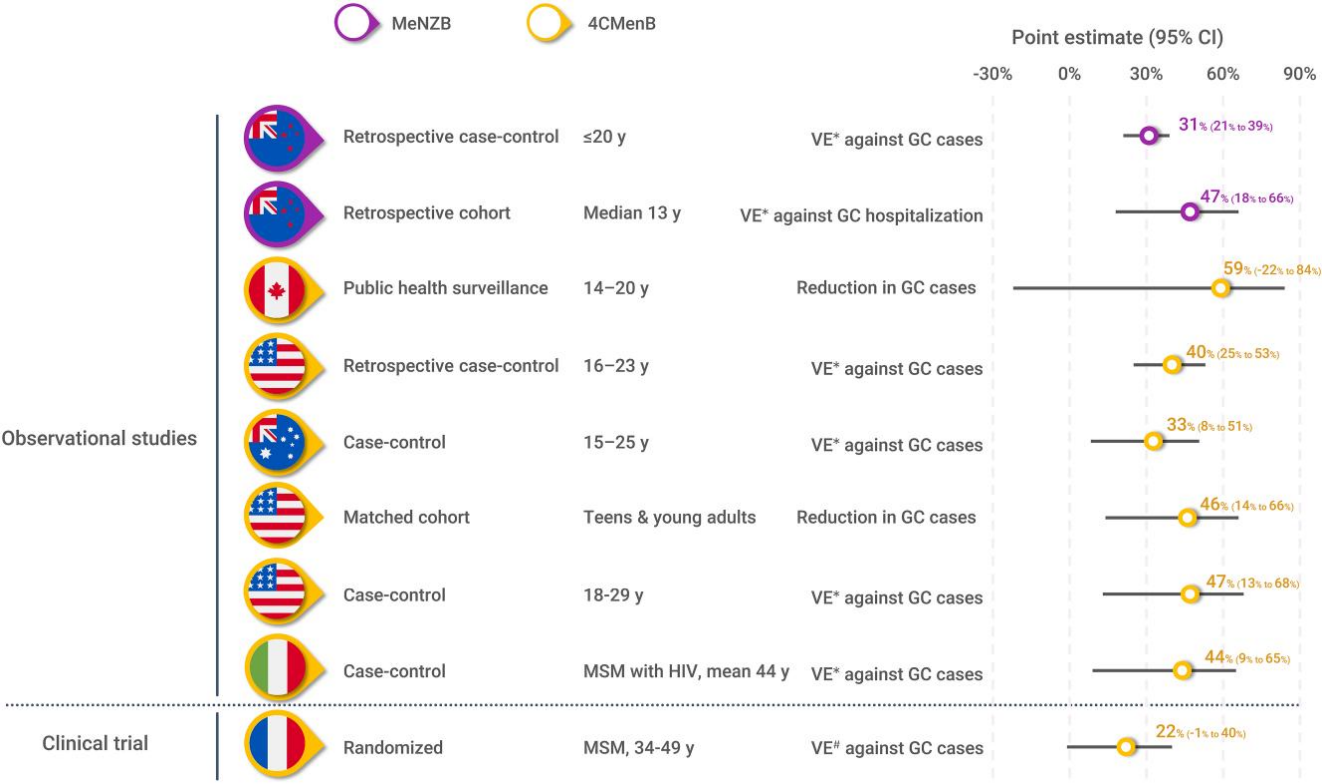
Current evidence for meningococcal group B outer membrane vesicle vaccines against *Neisseria gonorrhoeae.* Data from [31–38, 44]. Abbreviations: CI, confidence interval; GC, gonococcal; HIV, human immunodeficiency virus; MSM, men who have sex with men; VE*, vaccine effectiveness (observational study); VE^#^, vaccine efficacy (clinical trial).

There is currently no vaccine specifically targeting *N gonorrhoeae*. However, real-world evidence has suggested that a reduction in gonorrhea cases has been observed following vaccination programs against meningitis B (MenB), caused by *N meningitidis*. Data are available for 3 MenB vaccines containing outer membrane vesicles (OMVs): VA-MENGOC-BC, MeNZB, and 4CMenB. VA-MENGOC-BC is used in Cuba, MeNZB was widely used in New Zealand during an outbreak of MenB disease but is no longer available, and 4CMenB was first licensed in 2013 and is currently available in many countries. A comprehensive review article that summarizes the scientific and real-world evidence of MenB vaccines against gonorrhea has been published recently [30]. Several independent observational studies have provided evidence that *N meningitidis* group B–derived OMV vaccines (MeNZB and 4CMenB) are associated with a reduced risk of contracting gonorrhea (Figure 2) [31–38]. These results are subject to potential bias and limitations of observational studies. However, it is of interest to note that consistent results were reported across several studies assessing various geographies, populations, and using different methodologies. In addition, a modeling study of adolescent 4CMenB vaccination in England showed that vaccination could result in reductions in gonorrhea cases of 10%, 18%, and 25% over 10, 20, and 70 years, respectively, in individuals aged 13–64 years, based on 31% vaccine efficacy [39].

Supporting these findings, it has been shown in a female mouse model of *N gonorrhoeae* genital tract infection that immunization with 4CMenB speeded up clearance and reduced the burden of *N gonorrhoeae* compared with administration of placebo or alum [40]. Vaccination with 4CMenB induced serum IgG and vaginal IgG and immunoglobulin A (IgA) that cross-reacted with *N gonorrhoeae* OMVs [40]. Furthermore, antibodies from vaccinated mice recognized surface proteins from *N gonorrhoeae* including pili-associated protein Q and neisserial heparin binding antigen (NHBA) that are also detected in humans who have been immunized with 4CMenB [40].

In Cuba, a major reduction of cases of *N gonorrhoeae* was seen following introduction of VA-MENGOC-BC [41]. Intramuscular administration of VA-MENGOC-BC is known to induce a Th1 immune response characterized by induction of IgG and IgG1 but does not induce an IgA response [41, 42]. Vaccines containing aluminum hydroxide normally induce a Th2 immune response, but this pattern is changed to a Th1 response in OMV-containing vaccines [41]. Individuals vaccinated with VA-MENGOC-BC have serum IgG antibodies against *N meningitidis* proteoliposome (PL) but minimal IgA [41]. In carriers of *N meningitidis*, a booster dose of VA-MENGOC-BC induced significantly higher levels of mucosal anti-PL IgA compared with noncarriers, as well as IgG and IgA that were cross-reactive to several gonococcal proteins [41]. This suggests that natural carriage of *N meningitidis* in an immunized population might induce a mucosal immune response that impacts protection against acquiring gonorrhea [41].

**Figure 3.**
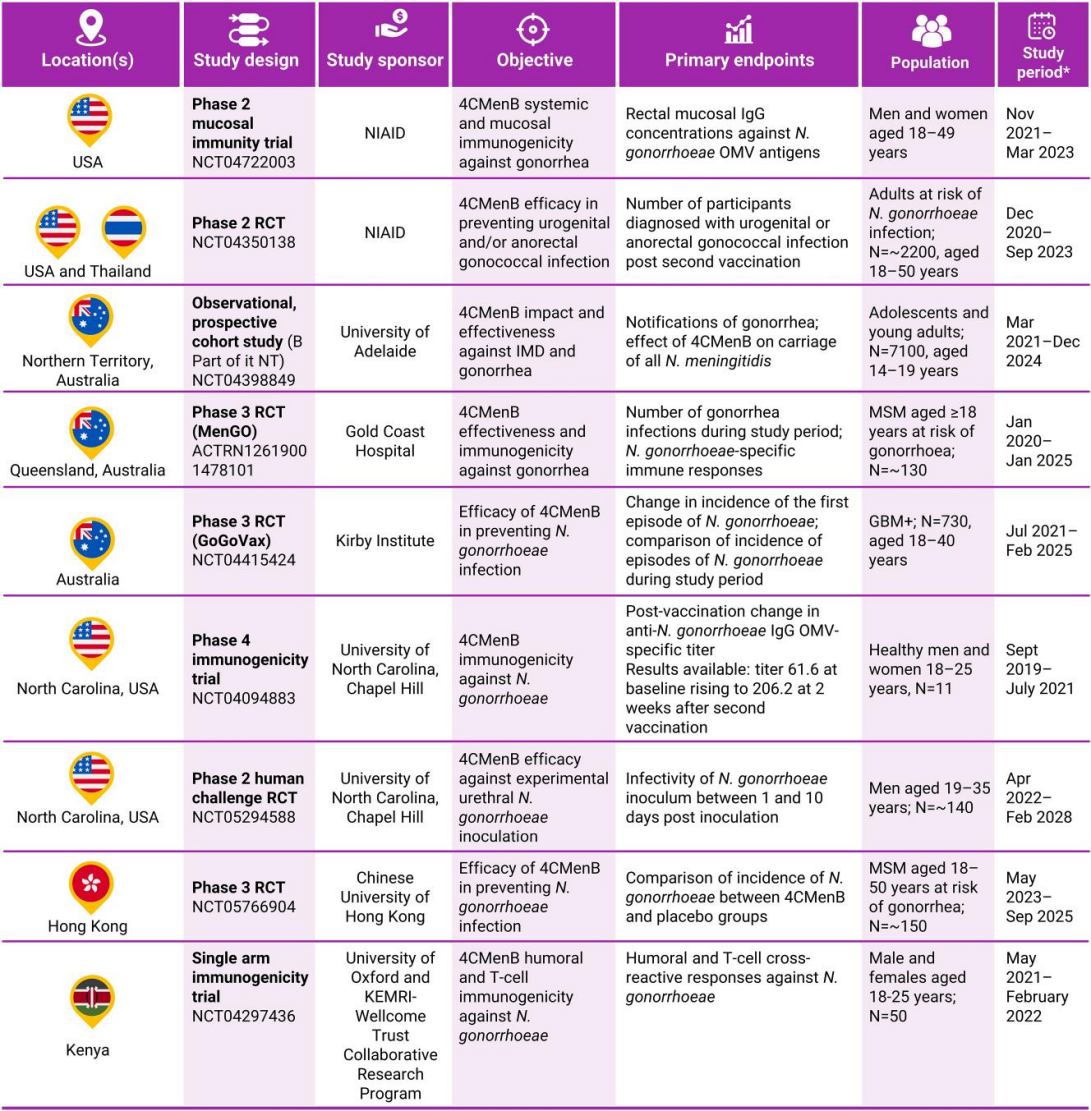
Ongoing clinical trials of 4CMenB vaccine against gonorrhea. *Study timelines subject to change. Abbreviations: ACTRN, Australian Clinical Trials Registration Number; GBM+, gay bisexual men+ (men [cisgender and transgender], transgender women, and nonbinary people who have sex with men); IgG, immunoglobulin G; IMD, invasive meningococcal disease; KEMRI, Kenya Medical Research Institute; MSM, men who have sex with men; NCT, National Clinical Trial; NIAID, National Institute of Allergy and Infectious Diseases; NT, Northern Territory; OMV, outer membrane vesicle; RCT, randomized controlled trial.

Several clinical trials of 4CMenB in prevention of gonorrhea are currently underway in countries including the United States, Thailand, Australia, Kenya, and Hong Kong (Figure 3). The first clinical trial to publish results was the National Agency for AIDS Research (ANRS) DOXYVAC trial, which assessed the effect of doxycycline postexposure prophylaxis (PEP) and 4CMenB against gonorrhea infection in men who have sex with men receiving PReP doxycycline therapy and with a history of an STI in the past 12 months. The results of the study were inconclusive. An interim analysis suggested a reduction in the number of cases of gonorrhea infection with both interventions, and the study was prematurely discontinued on the recommendation of the data and safety management board to offer doxycycline PEP and 4CMenB to all trial participants [43]. However, the final, postaudit analysis of the study reported that vaccine efficacy of 4CMenB against gonorrhea infection did not meet statistical significance (22% [95% confidence interval, −1% to 40%]; *P* = .061) (Figure 2) [44].

### Potential Mechanism and Role of 4CMenB Vaccine Components in Gonorrhea Prevention

The 4CMenB vaccine contains 3 main recombinant *Neisseria* protein antigens: *Neisseria* adhesin A (NadA), NHBA, and variant 1 of factor H binding protein (fHbp), which were selected based on their ability to induce serum bactericidal antibodies to homologous and heterologous MenB strains [45]. To increase immunogenicity, the accessory proteins, genome-derived *Neisseria* antigen (GNA) 1030 and GNA2091, are fused to NHBA and fHbp, respectively [46]. The vaccine also contains OMVs derived from a MenB outbreak strain (B:4:P1.7–2.4) from New Zealand, which is the same OMV component used in the MeNZB vaccine that also demonstrated a protective effect against *N gonorrhoeae* [31, 32]. OMVs contain several molecular moieties, but bactericidal antibodies are mainly produced against the porin protein, PorA [45].

**Figure 4.**
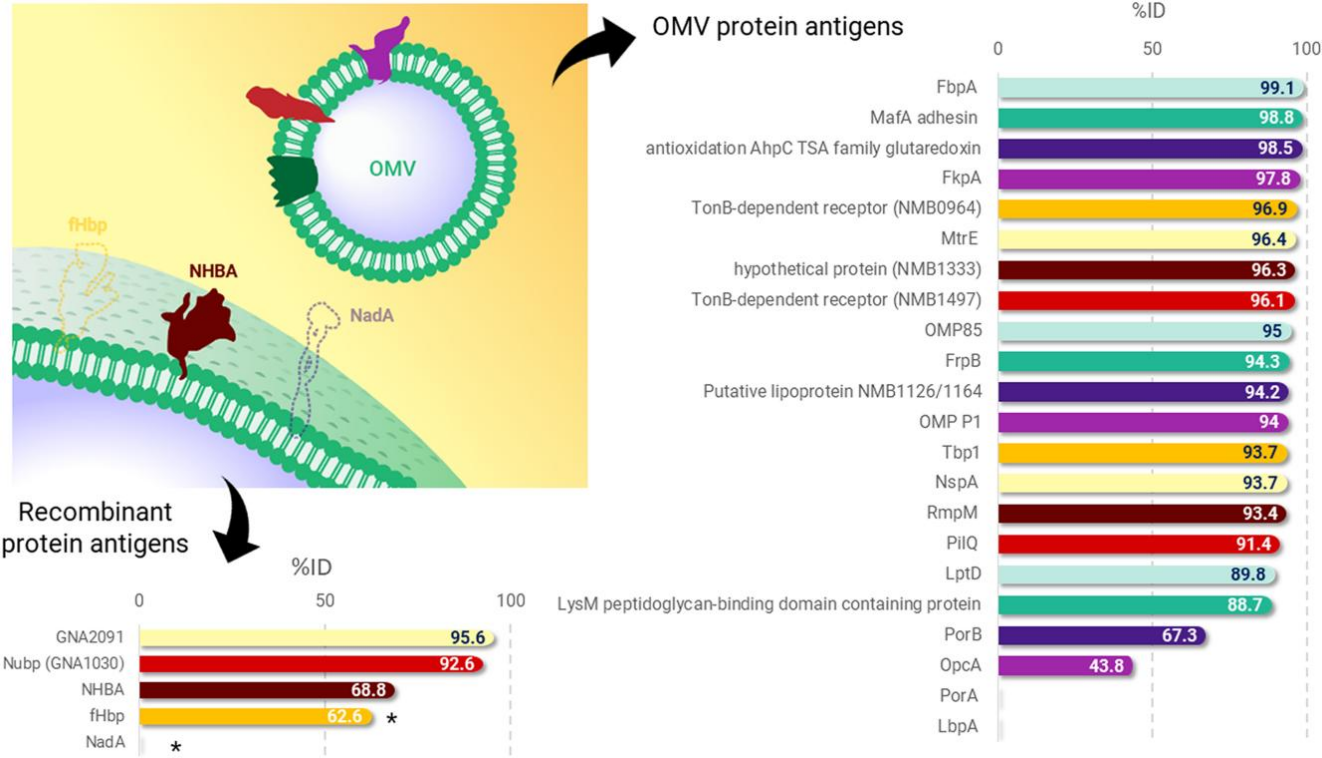
Homology of 4CMenB components to *Neisseria gonorrhoeae* proteins. From Semchenko et al [48]. *fHbp is not expressed on the gonococcal surface; NadA is missing in gonococcus. Abbreviations: %ID, percent identity; ABC, ATP-binding cassette; AhpC, alkyl hydroperoxide reductase C; FbpA, ferric binding protein A; fHbp, factor H binding protein; FkpA, FKBP-type peptidyl-prolyl cis-trans isomerase; FrpB, Fe-regulated protein B; LbpA, lactoferrin binding protein A; LptD, lipopolysaccharide-assembly protein; LysM, lysin motif; MafA, multiple adhesin family A; MtrE, outer membrane efflux protein; NadA, *Neisseria* adhesin A; NHBA, neisserial heparin binding antigen; NMB, *Neisseria meningitidis* strain MC58; NspA, *Neisseria gonorrhoeae* surface protein A; OMP, outer membrane protein; OMV, outer membrane vesicle; OpcA, opacity protein A; PilQ, pili-associated protein Q; PorA, porin, serosubtype P1.4; PorB, porin, major OMP PIB; RmpM, reduction modifiable protein M; Tbp1, transferrin binding protein 1; Tfp, type IV pili; TSA, thiol-specific antioxidant.

A study evaluating the prevalence of nucleotide and amino acid sequences of 4CMenB recombinant antigens in *N gonorrhoeae* isolates identified the presence of fHbp, NHBA, GNA2091, and GNA1030 genes but found that the NadA gene was absent [47]. The *N gonorrhoeae* strains exhibited 67%–95% identity in nucleotide sequence and 61%–95% identity in amino acid sequence to the 4CMenB GNA antigens [47]. However, truncated fHbp proteins were found in several isolates, resulting from premature stop codons in the genes [47]. A recent bioinformatic analysis also investigated the similarity of 4CMenB antigens to gonococcal proteins [48]. Of 22 core proteins in the OMV, 20 homologs were identified in *N gonorrhoeae*, of which 16 proteins had greater than 90% identity (Figure 4) [48]. The analysis also demonstrated that homologs of fHbp, NHBA, GNA2091, and GNA1030 are conserved in *N gonorrhoeae* but the gene encoding NadA is missing, confirming previous findings [48]. The analysis showed 68.8% identity of the NHBA antigen of 4CMenB to *N gonorrhoeae* (Figure 4) [48]. As fHbp, GNA2091, and GNA1030 are not surface-exposed on *N gonorrhoeae*, NHBA and OMV are likely the only antigens in 4CMenB that provide a protective effect against *N gonorrhoeae* [48].

It should also be noted that 4CMenB is administered via the intramuscular route and could therefore be expected to elicit a strong immune response that natural infection, with just mucosal contact, cannot induce.

## ONGOING STUDIES OF GONORRHEA VACCINE CANDIDATES

Several candidate vaccine antigens are being evaluated in preclinical studies (Table 1) [49–58]. NHBA, a component of 4CMenB as described earlier, is also being investigated as an antigen for a stand-alone gonorrhea vaccine. Anti-NHBA antibodies promoted complement activation and facilitated bacterial killing through both serum bactericidal activity and opsonophagocytic activity (Table 1) [49]. The antibodies also prevented the functional activity of NHBA by reducing heparin binding and adherence to cervical and urethral epithelial cells [49]. OMVs are also being investigated as platforms for a universal *Neisseria* vaccine, including an OMV with microencapsulated IL-12 as an adjuvant [50], an OMV modified to overexpress a mutant fHbp and an attenuated endotoxin [51], detoxified OMVs as immunogens [52], and an OMV genetically modified from a circulating gonococcal strain [53] (Table 1).

**Table 1.** Studies of Current Gonorrhea Vaccine Candidate Antigens in Preclinical Development

Description	Study Findings	Reference
Neisserial heparin binding antigen	Promoted complement activation, facilitated bacterial killing, reduced heparin binding, reduced adherence of NHBA to epithelial cells	Semchenko et al, 2020 [49]
Gonococcal OMV with microencapsulated IL-12	Faster clearance of gonococcal infection in mice compared with control. Generation of IgG and IgA antibodies against antigens from homologous and heterologous strains	Liu et al, 2017 [50]
Meningococcal OMV with attenuated endotoxin and overexpressing fHbp	Elicited human complement-mediated serum bactericidal antibody responses against a gonococcal strain	Beernink et al, 2019 [51]
Meningococcal detoxified OMV from strains lacking the major outer membrane proteins (PorA, PorB, and RmpM)	Enhanced gonococcal clearance in immunized mice. Clearance was associated with *Neisseria meningitidis* IgG antibodies that cross-reacted with *Neisseria gonorrhoeae*	Matthias et al, 2022 [52]
Two candidate vaccines based on a gonococcal OMV developed from recent Chilean strain and laboratory strain	Both candidates induced gonococcal-specific serum and mucosal IgG and IgA antibodies, as well as gonococcal-specific Th1/Th17 responses in mice. Both accelerated clearance of infection faster than 4CMenB	MacLennan et al, 2022 [53]
*Salmonella enteriditis* ghost delivering *N gonorrhoeae* PorB-based DNA vaccine	Elicited higher CD4^+^ and CD8^+^ T-cell responses and IgG responses compared with DNA vaccine alone in mice, along with a rise in bactericidal antibody titer	Jiao et al, 2018 [54]
*Salmonella enteriditis* ghost delivering *N gonorrhoeae N*spA-based DNA vaccine	Elicited high levels of anti-NspA antibodies and bactericidal antibodies in immunized mice; antibodies were highest in mice coadministered both the NspA-based and PorB-based vaccine	Jiao et al, 2021 [55]
Hybrid antigen of *N gonorrhoeae* TbpB used as a scaffold to display conserved surface epitopes of TbpA	Antisera raised in mice and rabbits against the hybrid antigens recognized surface TbpA of *N meningitidis* and inhibited transferrin-dependent growth	Fegan et al, 2019 [56]
Peptide mimic (mimitope) of LOS epitope 2C7 with Toll-like receptor 4 and Th1-stimulating adjuvant	Elicited bactericidal IgG, as well as reducing colonization levels and accelerating clearance of gonococci in infected mice	Gulati et al, 2019 [57]
Microneedle-based transdermal delivery of whole-cell inactivated *N gonorrhoeae* microparticles	Higher antigen-specific IgG antibody titers and antigen-specific CD4^+^ and CD8^+^ T-cells in mice versus gonococcal antigens in solution or empty microneedles	Gala et al, 2018 [58]

Abbreviations: fHbp, factor H binding protein; IgA, immunoglobulin A; IgG, immunoglobulin G; IL, interleukin; LOS, lipooligosaccharide; NHBA, neisserial heparin binding antigen; NspA, Neisserial surface protein A; OMV, outer membrane vesicle; Por, porin; RmpM, reduction modifiable protein M; Tbp, transferrin binding protein; Th1, T-helper 1 cell; Th17, T-helper 17 cell.

Bacterial ghosts are also being evaluated for use in gonococcal vaccines. Ghosts are empty shells of gram-negative bacteria that have an identical antigenic profile to the original bacterium and can be used for immunization with envelope antigens or as delivery vehicles for other molecules [59]. Ghosts have been used for delivery of an *N gonorrhoeae* PorB-based DNA vaccine and a neisserial surface protein A–based vaccine (Table 1) [54, 55]. The surface transferrin receptor proteins are important in the survival of *N gonorrhoeae* in the genitourinary tract of men and are therefore potential vaccine targets [56]. Transferrin binding protein B has been used as a scaffold to display conserved surface epitopes from transferrin binding protein A, which is an integral outer membrane protein [56]. The resulting hybrid antigen elicited protection against bacterial challenge in mice (Table 1) [56].

The 2C7 epitope of gonococcal LOS is conserved among gonococci and is immunogenic during natural infection, making it a potentially useful epitope as a vaccine antigen [60]. Immunization with 2C7-generated antibodies accelerated gonococcal clearance in infected mice (Table 1) [57]. A further approach being investigated is microneedle-based transdermal delivery of whole-cell inactivated *N gonorrhoeae* microparticles (Table 1) [58].

Only one candidate vaccine, the *N gonorrhoeae* generalized modules for membrane antigens vaccine, has reached a phase 1/2 clinical trial (NCT05630859). Potential next steps in the development of a gonococcal-specific vaccine are described in Box 1.

Box 1: **Next Steps in Development of a Gonococcal Vaccine**

**Table:** 

Ongoing trials of MenB vaccine in preventing gonorrhea can provide good infrastructure and opportunity for further research: Study of *Neisseria gonorrhoeae* Th17-driven responses and suppression of Th1- and Th2-driven responses Study of the immunogenetics of vaccinated individuals to understand and identify the immunodominant epitopes in the vaccine that explain the improved immune protection against gonococcal infection compared with naturally infected individuals Immune evasion in vaccinated individuals who acquire gonococcal infection Identification of correlates of protection
Confirm that the immunosuppressive pathways identified in mice are repeated in humans
Investigate Th1-driven responses induced by new vaccine candidates
Evaluate novel adjuvants and delivery systems to optimize mucosal responses to candidate vaccines
Adapt multiorgan cultures to combine primary human epithelial and immune cells to study human immune responses during infection
Further investigate the pathogenesis of *N gonorrhoeae* in the absence and presence of *Chlamydia trachomatis*

## CONCLUSIONS

The challenge for gonococcal vaccine development is that natural infection does not reliably induce immunity, and thus the classical approach of vaccine design, mimicking natural infection, is challenging. To develop vaccines with a superior protective immune response than that induced by natural infection, we need a better understanding of how *N gonorrhoeae* avoids inducing an effective immune response. Vaccines able to maintain Th1 response might be promising. However, work into potential candidate vaccine antigens is still at a very early stage and there is only one ongoing clinical trial in humans. Meanwhile, investigation of an apparent protective effect of MenB OMV vaccines is a high priority and clinical trials to further assess the effect of 4CMenB against gonorrhea are ongoing. Such trials should provide good infrastructure and opportunity for further research into immune responses and immune evasion of *N gonorrhoeae*.

## Notes

***Acknowledgments.*** The authors thank Erika Bartolini, Gaurav Mathur, Marta Palla, Ashley Broom, and Zeki Kocaata for their review of the manuscript. The authors also thank Business and Decision Life Sciences Medical Communication Service Center for editorial assistance and manuscript coordination, on behalf of GSK. Mary L. Greenacre (An Sgriobhadair, UK) provided medical writing services.

***Author contributions.*** Y. R. G. conceived this review. All authors participated in the interpretation of the data and the development of this manuscript. All authors had full access to the data and gave final approval before submission.

***Financial support*.** GlaxoSmithKline Biologicals SA funded this literature review and took in charge all costs associated with the development and publication of this manuscript including the Open Access publication charges.
